# Narrative Review of the Current and Future Perspectives of Phycobiliproteins’ Applications in the Food Industry: From Natural Colors to Alternative Proteins

**DOI:** 10.3390/ijms25137187

**Published:** 2024-06-29

**Authors:** Simeon Minić, Nikola Gligorijević, Luka Veličković, Milan Nikolić

**Affiliations:** 1 Department of Biochemistry and Center of Excellence for Molecular Food Sciences, Faculty of Chemistry, University of Belgrade, Studentski trg 12–16, 11000 Belgrade, Serbia; 2 Department of Chemistry, Institute of Chemistry, Technology, and Metallurgy, National Institute of the Republic of Serbia, University of Belgrade, Studentski trg 12–16, 11000 Belgrade, Serbia

**Keywords:** phycobiliproteins, food industry, natural colors, alternative proteins, C-phycocyanin, spirulina

## Abstract

Vivid-colored phycobiliproteins (PBPs) have emerging potential as food colors and alternative proteins in the food industry. However, enhancing their application potential requires increasing stability, cost-effective purification processes, and consumer acceptance. This narrative review aimed to highlight information regarding the critical aspects of PBP research that is needed to improve their food industry potential, such as stability, food fortification, development of new PBP-based food products, and cost-effective production. The main results of the literature review show that polysaccharide and protein-based encapsulations significantly improve PBPs’ stability. Additionally, while many studies have investigated the ability of PBPs to enhance the techno-functional properties, like viscosity, emulsifying and stabilizing activity, texture, rheology, etc., of widely used food products, highly concentrated PBP food products are still rare. Therefore, much effort should be invested in improving the stability, yield, and sensory characteristics of the PBP-fortified food due to the resulting unpleasant sensory characteristics. Considering that most studies focus on the C-phycocyanin from Spirulina, future studies should concentrate on less explored PBPs from red macroalgae due to their much higher production potential, a critical factor for positioning PBPs as alternative proteins.

## 1. Introduction

Phycobiliproteins (PBPs), pigment proteins from cyanobacteria and algae, are water-soluble multimeric proteins which form light-harvesting complexes called phycobilisomes. So far, there are sixty-one phycobiliprotein structures deposited in the Protein Data Bank [1], and all of them are divided into three groups: allophycocyanins, phycocyanins (including phycoerythrocyanin), and phycoerythrins [2]. PBPs are differently colored due to the presence of covalently bound chromophores called phycobilins. Phycobilins are tetrapyrrole molecules attached to the specific Cys residues of apoproteins via a thioether bond [1]. There are four types of phycobilins: phycocyanobilin (PCB), phycoerythrobilin (PEB), phycourobilin (PUB), and phycoviolobilin/crypto-violobilin (CVB) [2]. PBPs are hexameric proteins in their native and functional state with (αβ)_6_ structure. Allophycocyanin is the exception, where the functional unit is trimer (αβ)_3_ [3]. Each αβ monomer is composed of two subunits, α and β. Molecular masses of these subunits range from 16 to 17 kDa for α and 18 to 19 kDa for β. The number of bound chromophores per αβ monomer is associated with their division into three mentioned classes, with allophycocyanin having two chromophores, phycocyanins and phycoerythrocyanins having three, and phycoerythrins having five or six [4,5]. Within hexamers, two trimers are associated with face-to-face orientation, and one trimer is rotated by 30° relative to the other [6].

The presence of covalently bound phycobilins in PBPs provides them with a high potential for application in several areas, including the food and confectionery industry and biomedical applications [7]. Intensive colors allow these proteins to be used as natural pigments in the confectionery industry. Considering their high nutritional values [8], these protein sources are increasingly considered alternative food options. Phycobilins are also fluorescent, and this property is essential for their application as fluorescent biomarkers. Many recent review papers have covered these aspects [1,9,10,11,12,13]. Multiple biological activities of PBPs have also been investigated, and it is reported that they have antioxidant, anticancer, anti-inflammatory, antidiabetic, antibacterial, antiobesity, and neuroprotective properties [11]. Their bioactivity is also ascribed to covalently bound phycobilins, and, through digestion, bioactive chromopeptides can be released [14,15]. 

The primary sources of PBPs, cyanobacteria and red algae, are rich in protein content, with cyanobacteria Arthrospira platensis (commercially named Spirulina) having 55–70% protein in their dry weight [16], while red seaweeds range from 2.7 to 47% in dry weight, depending on the species [17]. PBPs are present in high abundance. Blue-colored PBPs can be obtained from cyanobacteria, like Spirulina, comprising up to 8–13% of dry biomass [18]. The sources of red and purple PBPs, like: Gracilaria spp. and *Pyropia*/*Porphyra* spp. (known as Nori), *Rhodella* spp., *Bangia* spp., and *Porphyridium* spp. yield fewer PBPs (e.g., [9]). On the other hand, the annual production of these species is about 50,000 tons for Spirulina [19] and much more for red algae, with China being the leading producer [20]. Considering that Spirulina gained a lot of attention due to its high nutritional value and many health benefits [20], most of the research regarding the applications of PBPs in food fortification or as food colors are conducted on Spirulina biomass or its main PBP, C-phycocyanin (C-PC). Much less research is accomplished on red algae and their PBPs, with new research emerging in the past several years. It should be noted that the high production of red algae (more than five million tons per year) gives these species massive potential for broader food applications, and they represent a good alternative food source [19]. 

Regarding human consumption, highly purified PBPs are not used, considering the high costs of final products due to the purification process. The purity requirements of PBPs for food usage are easily achievable and do not require expensive techniques [12], lowering the price of PBP-based food products. Biomasses containing PBPs are often used in dry powder, tablets, leaves, capsules, or liquid form [20]. These products can be considered as food supplements and not as alternative food. Fortification with PBPs or biomasses containing PBPs into other edible food products could have several benefits. First, it would increase the nutritional and bioactive values of other foods. Second, it would make seaweed and, thus, PBPs more acceptable to consumers. 

The critical aspect that should be considered in the field of alternative proteins is their allergenic potential. While the allergenicity of plant and insect proteins has been demonstrated in many studies [21], the allergenic potential of PBPs is still not explored, with only a few case studies indicating the allergenic potential of C-phycocyanin from Spirulina [22,23]. On the other hand, several studies suggested anti-allergenic properties of C-phycocyanin from Spirulina [24] and R-phycocyanin from red macroalgae *Porphyra haitanensis* [25]. Although further studies are required to explore PBPs’ allergenicity more comprehensively, current findings do not point to the high allergenic potential of PBPs, giving them a comparative advantage over insect and plant proteins. 

This narrative review article’s uniqueness is that it looks at the potential use of PBPs as alternative proteins. Fortifying existing food is one possible approach to consuming larger amounts of PBPs; however, this paper aims to show potential methods for obtaining new attractive food based exclusively on PBPs. To achieve this, it is necessary to solve several significant problems mentioned in the paper. Having PBPs used as food colorants is one thing compared to using them as alternative proteins in the diet. The first goal can be achieved with a few milligrams of PBPs per liter/kilogram [26]; the other requires larger amounts, bringing several obstacles that must be overcome. Those obstacles, regarding biomass cultivation, protein yield, purification with odor and taste issues, and stabilization of PBPs, are critically discussed in this review, together with the economic aspect for broader usage. The lower stability of PBPs needs to be overcome if PBP-based food products are to be made, especially using thermal treatment, and these stability issues and means to improve them are also discussed. 

**Figure 1:**
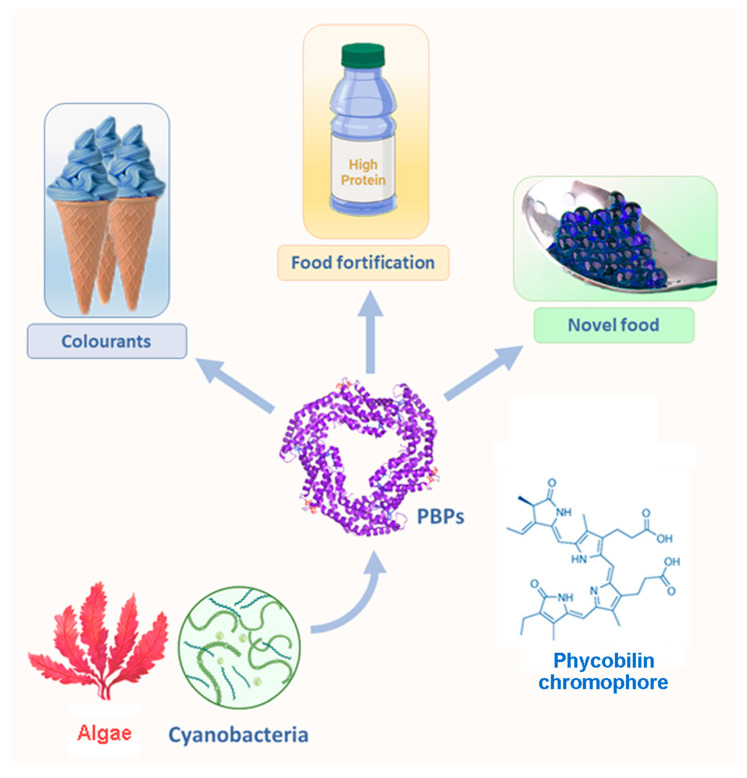
Overview of the applications of phycobiliproteins in the food industry.

Additionally, a detailed overview of current inventions in PBP-based food products, with technology to make them and fortification of the commonly used foods with PBPs, is presented and is the main focus of this review. Overall, the presented review paper should provide the reader with a solid literature overview of the current state-of-the-art application of PBPs as alternative food/protein sources (Figure 1). Ultimately, we proposed the future trends in the PBP science and industry domains that are required for positioning them as promising alternative proteins. 

## 2. Methods

The presented narrative review was realized by conducting a literature search, papers abstract, and full-text analysis, followed by discussions based on the results. The literature search was performed as described previously [27]. Relevant studies were obtained using the following databases: Google Scholar, Scopus, Web of Science, and PubMed. Papers were searched by combining several keywords, including phycobiliproteins, Spirulina, Nori, food fortification, stabilization, red algae, encapsulation, colors, etc. For this review to reflect recent, state-of-the-art progress in phycobiliprotein research in food application, most of the references included are no older than 5 years.

## 3. Phycobiliproteins as Food Colors

Color represents an essential characteristic of food and is a critical factor that makes food products more appealing to customers [28]. Considering that the general public is more interested in what they eat, natural food additives, including colors, are gaining more consumer and food industry interest [29]. Among colors, blue still represents a real challenge, and blue food is usually labeled by consumers as artificial [30]. The industry still uses artificial blue colors since natural ones lack physical and chemical stability, coloring power, and easy scale-up production [28]. The characteristic vivid coloration of PBPs makes them attractive in the food industry as natural food colors [31]. Their colors range from blue (C-PC), turquoise-blue (allophycocyanin), purple (R-phycocyanin), and red (R-phycoerythrin). Extract from Arthrospira platensis, rich in C-PC, is the only FDA-approved phycobiliprotein-based natural color [32], and it is also approved in the EU (EFSA regulation numbers 1333/2008 and 231/2012). It is approved for coloring confections, frostings, ice cream and frozen desserts, dessert coatings and toppings, beverage mixes and powders, yogurts, custards, puddings, cottage cheese, gelatin, breadcrumbs, and ready-to-eat cereals and as a color of hard-boiled eggshells. 

As with other natural colors, PBPs have certain limitations for broader usage. Based on stability studies of C-PC, for example, optimal storage conditions for this protein are in the dark with a temperature below 45 °C and at pH 5.5–6 [33]. This protein, on its own, without stabilizers, is sensitive to light, high temperature, and pH, meaning it cannot withstand many food processing techniques. Also, it cannot be efficiently used as a color for acidic drinks, as it is unstable under such conditions [34]. On the other hand, some results point out that acidic drinks, with pH around 3, can be colored with C-PC (concentration up to 0.11 mg/mL) and that the color was stable for 11 days when kept in the fridge [35]. C-phycocyanin was found to be a more versatile natural blue color than indigo and gardenia blue, as it gave a more acceptable light blue color in jelly gum and soft cover candy, as evidenced in exploratory studies [36]. 

R-phycoerythrin has a broader pH stability range, from 3 to 10 [37], but it is also sensitive to thermal treatment above 40 °C [38]. However, it was shown that high-pressure treatment of R-phycoerythrin solution at 450 MPa is much less detrimental for protein color than thermal treatment [39]. R-phycocyanin is stable in a pH range from 4 to 8 and has a melting point of about 53 °C [40]. These stability characteristics of PBPs are only guidelines, and specific characteristics will depend on the algal/cyanobacterial source. For instance, PBPs isolated from thermophilic organisms are much more stable at higher temperatures and have higher potential as food colors [41,42]. However, cultivating such organisms on a large scale is not achievable since they require much more energy. One important feature is the influence of PBPs purity on their stability. The thermal stability of food-grade C-PC was greater than that of reagent-grade C-PC [43].

For PBPs to be utilized as food colors to their full potential, stability issues must be overcome. Several approaches for stabilizing PBPs are published and covered in several recently published review papers, indicating the topicality of the problem [33,44,45]. They can be divided into (1) those involving stabilizing proteins by the addition of various additives (Table 1) and approaches using different processing methods to strengthen PBP stability, such as encapsulation, cross-linking, and high-pressure processing (Table 2). PBP stabilization is achieved by the addition of edible oils [46]; sugars, including glucose, sucrose, sorbitol, trehalose, fructose, etc. (e.g., [43,47,48,49,50]); proteins [51]; and other natural preservatives, like ε-polylysine [52] or cysteine [53]. Chemical modification of PBPs is also a possibility [54]. 

While many encapsulating strategies for the stabilization of PBPs exist [33,44,45], not all of them are acceptable for usage in the food industry, with some being more applicable in photovoltaic or pharmaceutical applications [67,78]. Encapsulation can provide higher storage, temperature, and pH stability because PBPs are immobilized into specific matrixes. Production of microcapsules of small size is fine if final products are to be used as carriers of active PBP components, for applications in pharmacy, for preservation and enrichment of other foods, or for use as food supplements. Higher concentrations and amounts of such products should be used as alternative protein sources. Utilizing biomass and/or raw extracts rich in PBPs in this regard is sometimes a more accessible and cheaper approach, but it can have certain limitations. Polysaccharide hydrogels are inexpensive to fabricate and are shown to be very efficient in improving the stability of incorporated PBPs. Another alternative would be to use protein-based gels. While they are more expensive, final products are more nutritional since they contain higher protein content. Using appropriate gelling conditions and/or cross-linkers is also very important as some may have adverse effects if eaten in larger amounts, like using sodium tripolyphosphate for cross-linking of water-soluble chitosan [79]. Considering that polysaccharide hydrogels are non-digestible and have no particular taste, additional enrichment in this direction is necessary for the product to be accepted by consumers. Incorporating PBPs in food products like cakes, ice creams, milk, bread, and cookies overcomes this problem since these products already have a rich and pleasant taste. In addition, by incorporating PBPs, they act as immobilizing matrixes, improving the stability of these proteins. The following section will summarize an overview of food products containing PBPs as alternative protein sources.

## 4. Phycobiliproteins as Alternative Proteins and Food Fortifiers

In the last decades, most research has focused on the various aspects of PBP bioactivities and the improvement of their color stability in the conditions described above. In terms of these studies, PBPs have been usually studied at lower concentrations (generally less than 0.2%). Although PBPs may contribute to the color and health-promoting activities of fortified food at these levels, their concentration is insufficient to influence the food structure and its nutritional properties significantly. On the other hand, there is a significant abundance of PBPs in algae; for example, the PBPs in red macroalgae *Porphyra* spp. contribute up to 3% [80], while the content of PBPs in cyanobacteria Spirulina could be more than 10% of the dry mass [81]. Therefore, there is a substantial potential for cyanobacteria and algae to be explored and positioned as promising sources of alternative proteins, with the PBPs as the key contributors. In this context, fortifying the food products with high concentrations of PBPs and their influence on the structure, techno-functional, and nutritional properties of foods could be more significant. In recent years, studies have focused on how PBPs (mainly C-PC from Spirulina) could change the structure and properties of the existing foods and develop new PBP-based products. Currently, products based on Spirulina are approved for use in the EU (Novel Foods Regulation (EC) 287/1997 (NFR 1997)) since its usage was widely present in EU territory before 1997, while approval is necessary for products from red microalgae novel food. 

### 4.1. Phycobiliprotein-Based Food Products

The development of PBP-based new food products is a new field in food science, and only several studies have been dealing with this topic in recent years. The PBP-based food may have significant potential since PBPs are the major proteins of cyanobacteria (e.g., Spirulina) and red algae (e.g., *Porphyra*), a source with the desirable amino acid profile, especially in terms of essential amino acid content, which is nearly equivalent to the egg albumin [17,82]. 

One promising approach is developing composite gels obtained from PBPs and algal-derived polysaccharides, which could enable the sustainable production of new foods. It was shown that the addition of polysaccharides such as ĸ-carrageenan and guar gum (from 0.1 to 0.4%) enhances the structure and influences the water-holding capacity of thermally induced C-PC gels (16%) through modulation of the protein secondary structure content as well as hydrophobic interactions and disulfide bonds [83]. 

**Figure 2:**
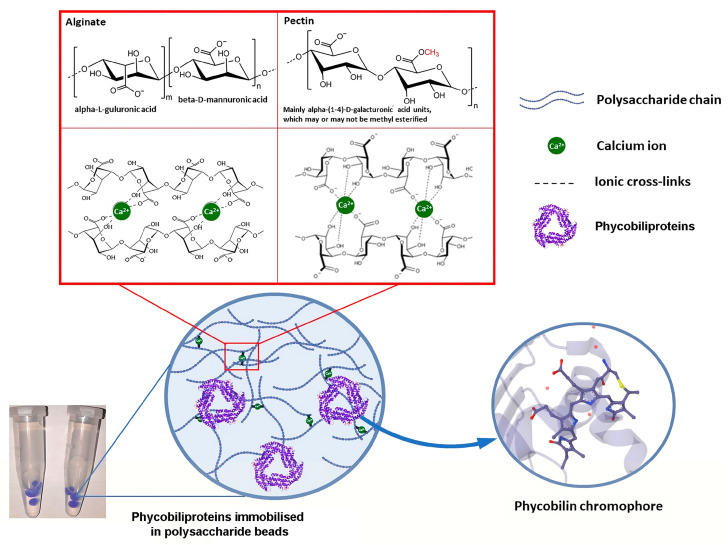
Encapsulation of phycobiliproteins into selected polysaccharide matrices [40].

Alginate, the well-known algal polysaccharide, has a significant application in the food industry due to its high propensity to make gels without organic solvents and at room temperature. Moreover, encapsulating proteins within an alginate gel network could significantly improve their stability, which is highly relevant regarding low PBP stability. Indeed, several studies revealed that the thermal stability of C-PC from Spirulina and R-phycocyanin from Porphyra sp. (Figure 2) could be significantly improved via PBP encapsulation within alginate beads [40,84]. Further, it was demonstrated that increased alginate concentration substantially enhances the encapsulation efficiency of C-PC. Alginate beads could not only improve the C-PC stability; they may also influence its bioavailability since the composite C-PC–alginate gel is resistant in the simulated gastric fluid, while rapid release occurs in the small intestine fluid medium [85,86]. Encapsulating R-phycoerythrin within alginate beads also improved its bioavailability at intestine-stage digestion [87]. On the other hand, C-PC, even at low concentrations (0.2%), can influence the properties of the alginate gels in terms of PC-containing beads containing more water structures with weak hydrogen bonds. 

Meanwhile, pregelatinized corn starch acts as a filler within alginate gels and absorbs surrounding water, which could improve the thermal stability of encapsulated C-PC. Pregelatinized corn starch enhances the encapsulation efficiency of C-PC within alginate beads [85]. Cross-linked starches from different botanical sources can encapsulate C-PC between amorphous chains. Among the five starches (potato, banana, corn, cassava, and breadfruit), potato starch exhibits the highest water uptake capacity and C-PC encapsulation efficiency [88].

Besides polysaccharide hydrogels, PBPs could also be encapsulated within protein-based gels. In terms of this, Spirulina extracts, containing a high amount of C-PC, improve the rheological and mechanical properties of soy protein isolate hydrogel (SPI) by enhancing its strength, hardness, and storage modulus. Furthermore, adding Spirulina extracts increases the content of β-sheet, which is why the hardness and compactness of the SPI hydrogel structure are improved [89]. Therefore, as the major component of Spirulina extracts, the C-PC could improve food texture. Another approach utilizes the interactions between C-PC and gelatin to form the self-assembly complex proteins. Due to the excellent rheological properties, this complex could stabilize high internal phase emulsions (HIPEs) used for the 3D printing of novel foods. The electrostatic interactions and hydrogen bonds between C-PC and gelatin enabled the compact structure, promoted the interfacial adsorption behavior at the oil–water interface, enhanced emulsion stability, and reduced the creaming index of HIPEs, followed by excellent extrudability. The 3D printing resolution and surface quality strongly depend on the C-PC content, and a concentration of 3.4% provides the best results regarding the compact intermolecular network system, the solid interfacial membrane, the improved mechanical strength, and the outstanding thixotropic properties [90]. Water-in-oil-in-water (W/O/W) double emulsions, composed of proteins and polysaccharides, were exploited to simultaneously and efficiently encapsulate hydrophobic astaxanthin and hydrophilic C-PC. The gelatin solution was used as an internal aqueous phase and soybean oil containing polyglycerol polyricinoleates was the oil phase, while sodium caseinate solution was utilized as an external water phase. The addition of gellan gum made the emulsion stable during the 30 days, and it can control the release of nutrients in the simulated digestion. Like alginate beads, W/O/W double emulsions were stable in a highly acidic stomach environment, with a strong protective effect on C-PC. In contrast, a weakly alkaline intestinal environment induced the release of nutrients [91]. Complexation with polysaccharides could significantly influence the digestibility and bioavailability of PBPs. In terms of this, the C-PC–chitosan complex stabilized emulsion showed better digestibility and oxidation stability than the free C-PC emulsion [92]. Moreover, besides alginate beads, alginate-based W/O/W double emulsion substantially improves the bioaccessibility of C-PC during intestinal digestion [93]. Besides the complexation with polysaccharides, lipid-based nano-carriers, such as Nano-phytosomes, are also used to improve C-PC’s gastrointestinal stability and bioavailability [94]. 

Recently, several studies used electrohydrodynamic processes such as electrospinning and electrospraying to encapsulate C-PC. These two processes represent facile, cost-effective, and flexible approaches that utilize electrically charged jets of polymer solution to produce fibers and particles at different scales. The significant difference between electrospinning and electrospraying is that the polymer solution concentration is much higher during the electrospinning process [95]. Incorporating C-PC from Spirulina into zein fibers via electrospinning resulted in packaging material that could protect walnut kernels from lipid oxidation. Furthermore, this approach enabled the high encapsulation efficiency of C-PC, and C-PC-loaded zein fibers were more active against pathogenic bacteria [96]. Electrospinning-induced co-encapsulation of C-PC and probiotics within the polyvinyl alcohol (PVA) nanofibers increases the antioxidant activities of the products. Moreover, the presence of C-PC within fibers improved the survivability of the probiotics [97]. C-Phycocyanin encapsulation in the PVA via electrospraying produced nanofibers with similar size and antioxidant activity properties. Thermogravimetric analysis in the same study demonstrated that encapsulation substantially increased the melting point of C-PC (from 59 °C for free protein to 216 °C for the encapsulated one) [98]. 

Phycobiliproteins not only promise bioactive and alternative proteins but also have significant potential to be used to produce alternative proteins in meat cultivation. Cultured meat is a promising solution to reduce land and water usage and limit pollution, but it is still costly. The critical challenge in cultivated meat science is to identify/develop fetal bovine serum (FBS) alternatives as growth supplements. Phycobiliprotein production is eco-friendly and cruelty-free; a relatively high concentration of PBPs in algae extracts could have significant potential to be positioned as FBS alternatives. The ability of C-PC-containing extract of cyanobacteria Anabaena sp. to partially replace FBS for the cultivation of muscle cell lines such as mouse embryonic myoblast line C2C12 and quail muscle clone 7 (QM7) cells was demonstrated. The same authors fabricated a 3D cell-dense structure by culturing QM7 cells in the same extract [99]. Another strategy comprises the incorporation of C-PC from Spirulina in chitosan/cellulose-based porous nanofilm, which enabled the controlled delivery of the protein in cell media for improving myoblast proliferation in a serum-reduced environment during long-term cultivation [100]. A similar approach was utilized to incorporate C-PC and growth factors in the edible fish gelatin microsphere. This was followed by their controlled release in a cell medium during myoblast cell cultivation at reduced serum conditions [101]. Although there has been some progress in the research of PBPs as potential FBS replacement components, a substantial workload in the future is required to develop the PBP-based media for the complete replacement of the FBS for meat cultivation.

### 4.2. Fortification of the Commonly Used Foods with Phycobiliproteins

**Figure 3:**
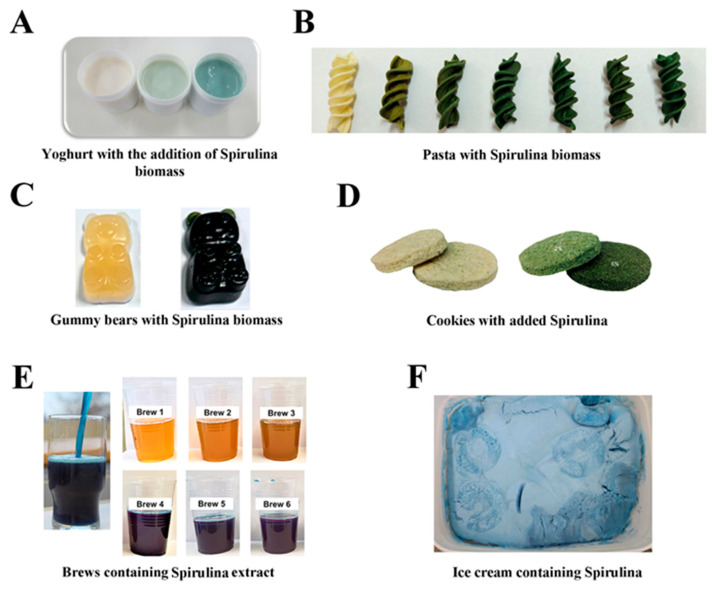
Selected examples of food fortification with the addition of Spirulina biomass or crude extract: (**A**) yogurt [103]; (**B**) fusilli pasta [104]; (**C**) gummy bears [105]; (**D**) wheat flour cookies [106]; (**E**) manufactured beers [107]; and (**F**) ice cream [108].

The research and industry activities could be more pronounced in developing novel food products based on PBPs as alternative proteins. On the other hand, numerous studies focus on fortifying commonly used food products with purified PBPs or PBPs containing algae extracts/biomass (Figure 3). Moreover, several food products (milk, ice cream, yogurt, juices, cookies, cheese, and candies) fortified with Spirulina extracts have appeared in the market in the last few years [102]. Table 3 summarizes the most significant findings about the effects of PBPs on fortified food in terms of texture, rheological properties, sensory acceptability, acidity, antioxidant activities, color stability, etc. It is important to emphasize that PBPs could have dual roles when added to commonly used foods: as food additives and the nutritive role as food ingredients. Although these two roles of PBPs are highly interconnected, PBPs at lower concentrations approach their “additive” role, while at higher concentrations, the nutrient aspect of PBPs becomes more pronounced. 

As seen in Table 3, most food products are fortified with Spirulina biomass or with the C-PC as the primary protein in Spirulina. Only a few studies are exploring the potential of red algae PBPs for coloring and food fortification. Considering the substantial annual production of red macroalgae *Gracilaria* spp. and *Poprhyra* spp. (around six million tons per year), fortifying food products with the PBPs from this source is promising [19]. It could be sustainable due to its much higher annual productivity than Spirulina, whose annual production is around tens of thousands of tons yearly.

The development of PBP-fortified food comprises the selection of optimal concentration to improve food texture, rheological properties, nutritional value, antioxidant activity, and color, but without hampering other sensory attributes and consumer acceptance. Adding PBPs or algae biomass improves fortified food products’ texture, rheological properties, and antioxidant capacity. Fortified products have higher protein content and improved emulsifying properties (Table 3).

Due to the fishy flavor of algae and cyanobacteria, one of the significant challenges to positioning PBPs from algae as food fortifiers is improving this sensory characteristic and, therefore, consumer acceptance [109]. Lower concentrations of C-PC or Spirulina biomass (up to 1%; *w*/*w*) have higher consumer acceptance than their higher content, with some exceptions (Table 3). Several strategies were employed to remove or reduce an unpleasant odor from algal biomass, such as adding sweeteners or aromas, which was covered in a recent review paper [110]. Another approach to avoid Spirulina’s unpleasant characteristic flavor is properly combining spices in the enriched food [111]. The type of Spirulina-enriched food also determines consumer acceptance; it has been shown that consumers from France, Germany, and the Netherlands have higher preferences for enriched pasta compared to enriched sushi and jerky [112]. Adding flavors to the Spirulina-enriched pasta, such as lemon-basil, improves consumer acceptance [113]. The response surface methodology was successfully implemented to optimize Spirulina-enriched soy yogurt’s ingredient formulation, processing parameters, and sensory parameters [114]. By optimizing the high moisture extrusion technique, it was possible to substitute soy partly with Spirulina biomass, producing firm and fibrous soy-based meat alternative products with a decent algae flavor [112]. This technique also shows promise for Spirulina extracts since mixing with lupin proteins can create meat analogs with improved physicochemical and nutritional properties [115]. Microencapsulation of Spirulina biomass in maltodextrin and Arabic gum could mask the taste of seaweed and seaweed aroma in the ice cream [116], while microencapsulation of Spirulina in alginate spheres resulted in higher consumer acceptability [117].

**Table 3:** Overview of using natural sources of vivid-colored phycobiliproteins in the food industry to fortify the commonly used food products.

Protein Sample	Concentration (*w/w*)	Food Type	Major Food Product Characteristics	Reference
Phycoerythrin-rich water extract (*Porphyridium cruentum*)	0.00015–0.00029%	Commercial beverages (e.g., gin and wine)	The pink color was stable during 11 days of storage; well accepted by a semi-trained panelist	[26]
C-PC from Spirulina (*Arthrospira platensis*)	0.025%	Ice cream	Stable color during the 6 months; increased antioxidant activity after in vitro simulated digestion	[108]
PBPs from cyanobacteria *Nostoc* sp.	0.03–0.14%	Skim milk	Satisfactory sensory characteristics	[118]
Encapsulated R-phycoerythrin (*Kappaphycus alvarezii*)	0.1%	Ice cream	Better rheology; pink color intensity increased during 90 days of storage; enhanced antioxidant activity	[119]
C-PC from Spirulina (*Arthrospira platensis*)	0.1–0.2%	Ice cream	Smoother and softer texture; sugar (25%) and fat (50%) content reduction; no significant influence on consumer acceptance	[120]
*Porphyridium cruentum* spray-dried biomass	0.1–0.3%	Ice cream	Increased consistency index	[121]
C-Phycocyanin extract	0.18–0.32%	Soft beverage	Improved product’s antioxidant activity; good sensorial attributes	[122]
Spirulina (*Arthrospira platensis*) biomass	0.25%	Craft beer	Increased total polyphenols, tannins, and antioxidant power; cytoprotective properties towards the oxidative damage	[123]
Spirulina (*Arthrospira platensis*) powder	0.25–1%	Yogurt	Better water holding capacity and lower whey syneresis (28 days of storage); improved antioxidant activity; lower firmness but better elasticity; acceleration of the end of fermentation; acceptable sensory characteristics only at 0.25%	[124]
C-Phycocyanin	0.3%	Biscuit	High oxidative stability during 30 days of storage; satisfactory all main sensory characteristics (e.g., odor, flavor, texture, and overall acceptability)	[125]
C-PC from Spirulina (*Arthrospira platensis*)	0.3–0.4%	Ice cream	Emulsifying and stabilizing activity; lower consumer acceptance	[126]
Spirulina (*Arthrospira platensis*) powder	0.4–1.2%	Bread	Higher moisture content; lower hardness; highest consumer acceptance with 0.8%; higher antioxidant activity	[127]
Spirulina (*Arthrospira platensis*) microencapsulated in alginate	0.5%	Yogurt	Improved viscosity stability during storage; better consumer acceptance	[103]
Spirulina (*Arthrospira platensis*) powder	0.5–1.5%	Feta-type cheese	A higher number of lactic acid bacteria after 60 days of storage; softer texture and sensory characteristics (at 0.5 and 1%)	[128]
C-PC from Spirulina (*Arthrospira platensis*)	0.5–2%	Cow’s milk	Increased solid non-fat content; enhanced antioxidant activity; improved sensory characteristics	[129]
*Spirulina maxima* biomass	0.5–2%	Pasta	The color was relatively stable after cooking, with increased firmness. Higher consumer acceptance scores	[130]
Spirulina (*Arthrospira platensis*) powder	0.5–3%	Processed cheese	Decrease in adhesiveness, cohesiveness, springiness, chewiness; increase in hardness and gumminess; deterioration in the overall sensory acceptability	[131]
Spirulina (*Arthrospira platensis*) powder	0.63–2.5%	Fresh noodles	Increased antioxidant capacity; increased hardness, cohesiveness, springiness, gumminess, and chewiness; the highest consumer acceptance with 1.25%	[132]
Spirulina (*Arthrospira platensis*) powder	1%	Yogurt	Higher antioxidant activity and number of lactic acid bacteria; higher water holding capacity and viscosity; lower syneresis; decreased consumer acceptance	[133]
Spirulina (*Arthrospira platensis*) biomass and wheat germ	1% (both)	Pear–cantaloupe-based beverage	Increased antioxidant capacity, total phenol, and flavonoid content; good organoleptic score	[134]
Spirulina (*Arthrospira platensis*) powder	1.5–3.5%	Yogurt spread	Increased viscosity and spreadability; lower consumer acceptance with a higher Spirulina concentration	[135]
Spirulina (*Arthrospira platensis*) powder	1.6%	Baguette bread	Decreased hardness and gumminess; lower sensory score	[136]
Spirulina (*Arthrospira platensis*) powder	1–15%	Gluten-free fresh pasta	Higher antioxidant activity, without affecting product cooking and texture quality properties; a favorable sensory evaluation	[137]
Spirulina (*Arthrospira platensis*) biomass	1–2%	Cookie	Harder and darker product with increased protein content; questionable sensory quality	[138]
Spirulina (*Arthrospira platensis*) extract	1–5%	Chinese-style pork-sausage	Small changes in pH; inhibition of lipid oxidation; 2.5 and 5% retarded the decrease in sensory acceptability (storage at 4 °C)	[139]
Spirulina (*Arthrospira platensis*) biomass	2 or 6%	Cookie	Color and texture stability over 8 weeks; higher protein and total phenolic content and in vitro antioxidant capacity; without in vitro digestibility changes	[106]
Spirulina (*Arthrospira platensis*) biomass	2%	3D-printed cookie dough	All formulations were suitable for extrusion and microbiologically stable; stable texture after 30 days of storage; improved antioxidant properties and color stability after extract encapsulation in alginate microbeads	[140]
Spirulina (*Arthrospira platensis*) powder	2.5–10%	Pasta	Increased rheological parameters, color, and cooking quality; decreased dough stability; sensory acceptable up to 5%	[141]
Nano-liposomes containing PBPs from *Gracilaria gracilis*	2.5–5%	Carp burger	Lower oxidative spoilage and microbial deterioration; no significant loss of overall consumer acceptability (18 days of refrigerated storage)	[142]
Spirulina (*Arthrospira platensis* F&M-C256) biomass	2–10%	Sourdough bread	Higher antioxidant activity; highest consumer acceptance with 2%	[143]
Spirulina (*Arthrospira platensis*) biomass	2–15%	Pasta	Lower firmness, cut force, and consistency; higher stickiness; highest consumer acceptance for 12.5%	[104]
C-PC from Spirulina (*Arthrospira platensis*)	2–8%	Yogurt	Decreased syneresis; increased firmness and viscosity; higher pH and color stability; no pathogen growth during 21 days of storage; overall acceptability not affected at 4%	[144]
Microencapsulated Spirulina (*Arthrospira platensis*) in alginate	3%	Pasta	Protection of antioxidant potential; higher firmness; acceptable sensory characteristics	[117]
Spirulina (*Arthrospira platensis*) powder	4–6.5%	Dry noodles	Lower cooking loss; higher elongation and tensile strength; highest consumer acceptance with 6%	[145]
Spirulina (*Arthrospira platensis*) powder	5%	Beer	Slightly altered fermentation parameters; typical beer-like product character; odor and taste alteration compromising the consumer acceptance	[107]
Microencapsulated *Spirulina* sp. LEB-18 in maltodextrin and soy lecithin	5–8.75%	Chocolate milk	Increased antioxidant activity; good suspension stability and low hygroscopicity; questionable consumer acceptance.	[146]

### 4.3. Challenges for Proper Phycobiliproteins Utilization as Alternative Proteins

Several challenges must be addressed and overcome to enable a broader utilization of PBPs as natural colorants and alternative food proteins, mainly if the entire biomass or raw extracts are to be used. These can be divided into issues considering biomass cultivation, safety concerns, and bioavailability of PBPs, as well as their quantity and purity. Connected with these are the economic aspects of the high prices of PBPs.

#### 4.3.1. Cultivation of Cyanobacteria/Algae as Sources of Phycobiliproteins

Some considerations regarding the cultivation risks of cyanobacteria/algae also need to be addressed. They include contamination risks and adverse effects on biodiversity. 

Edible seaweed may contain several contaminants, including heavy metals, elevated iodine content, anti-nutritional factors, radioactive isotopes, ammonium, dioxins, and pesticides. However, the safety of seaweed largely depends on the cultivation environment [17,147]. Heavy metal contamination represents the highest risk factor. Biomasses of cyanobacteria and microalgae are known to assimilate toxic heavy metals during their growth due to the presence of alginic acid, proteins, and peptidoglycans present in the extracellular matrix [17,148]. Commercially available seaweed samples were shown to contain As, Cd, and Pb [149]. Cyanobacterial species like *Microcystis aeruginosa* can result in biomass having toxic microcystins [150], which is a significant issue if larger quantities of PBPs are to be used as alternative protein sources. Therefore, it is imperative to implement proper quality control measures that prevent the production of toxic biomass for human nutrition. 

Another concern that should be addressed is the potential allergenic properties of cyanobacteria and algae as sources of phycobiliproteins. However, there are limited findings regarding the allergenic properties of PBP-producing cyanobacteria, and only a few case studies about Spirulina and C-PC allergenicity were reported and classified as anaphylaxis [151]. Furthermore, studies addressing other health issues of PBPs in animals and humans are quite scarce. Until now, it was demonstrated that the C-PC from *Galdieria sulphuraria* [152] and Spirulina [153] did not induce any toxic effects in rats and humans, respectively. 

Growing particular algae species in large quantities can negatively affect marine ecosystems [154], as they can compete with other species. There is a risk for extreme proliferation and invasion of non-native species when conditions for their overgrowth are met. For example, in the case with China in 2008, a massive green tide occurred, covering about 600 km^2^ along the coast of Qingdao [155], which is one of the reasons why algae cultivation needs to be strictly controlled. While macroalgae may enhance biodiversity [156], their subsequent harvest, if not properly managed, may damage this habitation and thus negatively affect other species living there [157]. Marine organisms that live below the level of seaweed and are dependent on sunlight can also be negatively affected by mass production of algae due to lower irradiance [154].

#### 4.3.2. Isolation and Purification of Phycobiliproteins from Cyanobacterial/Algal Biomass

Once biomass that passes safety regulations for human consumption is obtained, the next obstacle is getting PBPs in sufficient quantity and quality. The purity index of PBPs is expressed as the ratio of absorbance at wavelengths of maximal adsorption in the visible part of spectra and absorbance at 280 nm. In the case of C-PC, for example, food-grade protein has an A_620 nm_/A_280 nm_ ratio of 0.7, reagent grade has a ratio of 3.9, and analytical grade has a ratio over 4 [158]. So far, several review papers and book chapters have already covered many published papers about the isolation and purification of PBPs from many algal sources [1,9,33,159,160]. After obtaining protein extracts by simple solvent extraction from biomass or additional treatments, including sonication, nitrogen freezing, lyophilization, or addition of enzyme(s), the next step is the purification of desired PBPs. While standard chromatography techniques give highly purified PBPs, the yield is usually meager. Additionally, they are expensive and complicated to implement for broader usage of PBPs as alternative food proteins. Therefore, other cheaper methods that can be easily scaled up to obtain sufficient quantity and quality PBPs are required. Promising methods are aqueous two-phase separations [161,162], membrane chromatography/technology [163,164], expanded-bed adsorption chromatography [165], and ultrafiltration [166,167]. All of them can yield more and provide sufficient purity of PBPs for food applications.

#### 4.3.3. Economic Aspects of Phycobiliproteins Production

One of the significant obstacles in positioning PBPs as competitive alternative proteins is their high price, mainly due to the costly cultivation of PBP-containing cyanobacteria and algae. The price of Spirulina is usually in the range of EUR 30–70 per kg in Europe [168], but in the Asian market, the Spirulina prices could be much lower (EUR 5 per kg or higher). The price of food-grade C-PC also exhibits significant variations on the market. Generally, it is around EUR 100 per kg [169], which is still costly to establish as an alternative protein. The second significant challenge, closely related to the high C-PC price, is the relatively low production of Spirulina, which is around 50 thousand tons per year according to the report of the Food and Agriculture Organization of the United Nations (FAO) for the 2019 year. This amount may satisfy the market in the context of C-PC as food colorants, but translating PBPs to alternative proteins requires much higher productivity. Therefore, the increase in the Spirulina cultivation fields and the reduction in the price of cultivation are prerequisites for positioning C-PC as a competitive alternative protein. The cheap alternative culture media for growing Spirulina using industrial and processing wastes could be a promising approach to decrease the cultivation costs [170]. Cultivating *Spirulina maxima* in wastewater from the demineralization of cheese whey would lower production costs by 50% [171]. While utilizing the concept of the circulatory economy could significantly reduce the price of the final products, some challenges, such as the uncertainty of nutrient content and toxicity, should be addressed regarding Spirulina cultivation in wastewater [172]. The second, circulatory economy-based approach utilizes the CO_2_ from flue gas of biomass power plants and media recycling to reduce the cost of nutrients for *Spirulina maxima* cultivation by up to 42% [173]. Moreover, the exploitation of the biorefinery approach, which comprises the use of residual biomass after C-PC and high-value metabolites extraction as a potential source for the production of low-value, high-volume biofuels, could also be a promising approach for Spirulina and C-PC price reduction [174].

The most economically critical red macroalgae, such as *Porphyra* spp. and *Gracilaria* spp., have substantially higher annual production than Spirulina, and over 5 million tons of these algae are produced annually (FAO report). Although they have nearly one order of magnitude lower PBP content, their high annual production can be a source of alternative proteins [17]. Despite the high yearly production, the price of *Porphyra* spp. is still high, ranging from around EUR 100 per kg in the European market. Meanwhile, in the Asian market, the price starts at around EUR 5 per kg. The *Gracilaria* spp. has a similar price on the European market [168], while the prices on the Asian market may decrease to EUR 1 per kg for high-quantity purchases. However, these prices are still higher than those of the plant protein source [17]. On the other hand, the vivid and strong bioactive effects of PBPs could receive higher consumer acceptance compared to other sources of alternative proteins. There are no food-grade PBPs from red algae on the market; they can only be purchased as expensive research reagents for fluorescent labeling.

Contrary to C-PC from Spirulina, PBPs from red macroalgae are more challenging to extract, representing the obstacles in delivering them to the food market as colorants and alternative proteins [17]. Therefore, future studies are required to improve the extraction yields of PBPs (and other proteins) in cost-effective and food-compatible manners from red macroalgae and position them in the food market. Moreover, significant efforts could be made to improve the flavor characteristics of red algae extracts.

## 5. Conclusions and Future Perspectives

To date, significant efforts and progress have been made in the context of PBP purification and characterization. C-PC is currently the best-studied PBP, a natural and bioactive colorant for various foods and beverages. However, enhancement of its application potential in terms of availability on the market and competitiveness requires additional steps: (1) increase in Spirulina’s annual productivity, (2) improvement of C-PC stability using approaches compatible with food safety, and (3) minimizing or completely removing the unpleasant flavor characteristic arising from Spirulina. Considering the high abundance of C-PC in Spirulina, future studies should also focus on developing new, C-PC-based and protein-rich foods. These products would significantly strengthen the potential of C-PC as an alternative protein. Moreover, the vivid blue color of C-PC and its substantial bioactive properties could give it a significant advantage in the alternative protein market compared to other sources of alternative proteins. 

Phycobiliproteins from other sources are much less explored compared to C-PC from Spirulina. Considering the annual productivity of red macroalgae (such as *Porphyra* and *Gracilaria*) is more than two orders of magnitude higher than Spirulina’s yearly yield, there is a vast potential for these algae to be valorized as the source of PBPs for food application. However, establishing the PBPs from red macroalgae as food colorants and alternative proteins in the first place requires substantial research efforts to improve their extraction yield and characterization and enhance their stability. The following steps should also include the improvement of their sensory characteristics.

Based on the presented review, there are several obstacles for the food industry to overcome if phycobiliproteins are to be used more effectively and significantly. The main goals of the food industry regarding a particular product are customer satisfaction, safety, providing product information, and the maintenance of commercial viability. Developing high-quality (in terms of both nutritional and sensory characteristics, but also in terms of their safety) PBP food products, either as novel foods or as fortification in existing food products, would significantly improve consumer acceptance and satisfaction. This achievement will trigger the high demand for PBPs in the food market, which could be a motivation for the higher productivity of cyanobacteria and algae as sources of PBPs. Implementing the concept of a circulatory economy in the production of cyanobacteria and algae could enhance their productivity and decrease the cultivation price, creating a stronger position for PBPs as alternative proteins in the competitive food market by maintaining the commercial viability of PBP-based products. 

## Figures and Tables

**Table 1:** Effects of additives on stabilities of phycobiliproteins.

Protein (Source)	The Experimental Approach	The Results	Reference
C-phycocyanin (*Arthrospira platensis*)	Color stabilization of protein via λ-carrageenan (λC) in liquid formulations	Complexation with λC increased the protein color stability at pH < pI, especially at a pH of 3.0, even when heated to 90 °C.	[55]
C-Phycocyanin (*Arthrospira platensis*)	Stabilization effects of protein complexation with λ-carrageenan on its intrinsic blue color	The electrostatic complexation stabilized protein color in the acidic pH range (2.5–6.0) and against a heat treatment at 70 °C.	[56]
C-Phycocyanin (*Spirulina platensis*)	Stabilization of protein in aqueous solutions via sucrose and trehalose (20 and 40%, *w*/*w*)	The stabilizing effect of saccharides on the thermal discoloration of protein with sucrose performed better than trehalose.	[43]
C-Phycocyanin (Hawaiian Spirulina)	Investigation of the potential of twelve food-derived antioxidants to bind and stabilize the protein	Complexation of protein with quercetin and coenzyme Q_10_ improved its thermal stability (higher melting point).	[57]
C-phycocyanin (*Arthrospira platensis*)	Enhancement of protein productivity and stability using organic acids (citric, acetic, succinic, fumaric, and oxalic acid)	Organic acids, primarily citric acid (7.5%), act as preservatives to stabilize protein (promoting the half-live) at high temperatures.	[58]
C-phycocyanin (*Arthrospira platensis*)	Improvement of protein stability by adding saccharides (glucose, mannose, galactose, and maltose) and sugar alcohols (mannitol and maltitol)	Sugars effectively improved the protein’s thermal stability in correlation with the additive concentration and inhibited its oxidative degradation.	[59]
C-phycocyanin (*Spirulina platensis*)	Increasing protein stability via 0.5% cysteine addition during enzyme-assisted extraction	Cysteine increased the thermal stability of protein extracted with (endopeptidase) Collupulin.	[53]
C-phycocyanin (Spirulina)	Improvement of protein stability by forming soluble complexes with poly-saccharides (κ-/ι-/λ-carrageenans, xanthan gum, high-methoxyl pectin, and guar gum)	Improved protein’s colloidal and color stabilities against acidic pH (standard beverage processing) and heating conditions.	[60]
R-phycoerythrin (*Porphyra haitanensis*)	Stabilization of protein by self-assembly with oligochitosan (at a 1:20 reaction ratio)	The thermal (40–80 °C), natural light, and ultraviolet light irradiation (254 nm) protein stabilities were all improved.	[61]
Phycobiliproteins (*Oscillatoria* sp. BTA-170)	Stabilization of C-PC, A-PC, and PE in the presence of different monosaccharides (glucose, fructose, glucose, and lactose)	Glucose was the most critical monosaccharide that stabilizes the degradation of proteins at 65 °C and higher temperatures.	[62]
Phycobiliproteins (*Spirulina platensis*)	More efficient extraction of protein using NaCl as an extraction enhancer	Protein stability was improved by adding NaCl, which had unaffected antioxidant activity and a secondary structure.	[63]
C-Phycocyanin	Improving the protein color stability with epigallocatechin gallate (EGCG)	EGCG binding protected protein against color fading under light conditions.	[64]
R-phycocyanin (*Cyanidioschyzon merolae*)	Preservation of thermotolerant protein during storage with salts	The stabilizing effect of CaCl_2_ and MgCl_2_ (0.1 M) towards protein during seven days.	[65]
C-Phycocyanin	Improving the protein color stability in acidified conditions with whey protein isolate (WPI)	A low WPI concentration (0.05–0.1%) at pH 3.0 improved the protein’s color stability under light exposure.	[66]
C-phycocyanin (Spirulina)	Improvement of protein stability in acidified conditions using whey proteins (α-lactalbumin, β-lactoglobulin, BSA, immunoglobulins, and glycomacropeptides)	Native whey protein (10%) efficiently improves protein colloidal stability and prevents aggregation at pH 3.0.	[51]

**Table 2:** Processing methods for phycobiliprotein stabilizations.

Protein (Source)	Method and Conditions Used	Result	Reference
R-Phycoerythrin (*Gracilaria gracilis*)	Protein incorporation into the gelatin-based films	Improved the protein photochemical stability in the solid state for eight months.	[67]
C-Phycocyanin (*Arthrospira platensis*)	Preparation of pectin–phycocyanin complexes with different mixing ratios	Improved the colloidal stability of the protein wholly entrapped by the polysaccharide molecules at acidic pH after heating at 85 °C.	[68]
C-phycocyanin (*Spirulina platensis*)	Double encapsulation of protein using aqueous two-phase systems (PEG 4000/Potassium phosphate and PEG 6000/Dextran) by spray drying	Prolonged shelf life with the additional benefit of enhancing the purity of protein compared with conventional (maltodextrin) encapsulation.	[69]
Phycobiliproteins (*Spirulina platensis*)	Proteins were treated with high hydrostatic pressure (HPP) (600 MPa; 300 s) in the presence of sucrose, trehalose, and glucose (20 and 40%, *w*/*w*)	Sugars exerted baroprotective, concentration-dependent action on proteins’ (color) stability with preserved antioxidant activity.	[70]
C-Phycocyanin (*Arthrospira platensis* EGEMACC 38)	Spray-dried microencapsulation of protein using various combinations and ratios of wall materials (maltodextrin, gum arabic, whey protein isolate, and sodium caseinate)	The highest blueness index was observed in protein powder encapsulated with maltodextrin and whey protein isolate.	[71]
C-phycocyanin (Spirulina)	Modification of protein with 20 kDa methoxy polyethylene glycol polymers	The conjugates exhibited higher blue color intensity, improved thermodynamic stability, and a gain in pH stability and antioxidant activities.	[72]
Phycocyanin and B-Phycoerythrin	Intercalation of proteins into montmorillonite and laponite laminar nanoclays	Proteins’ optical and thermal properties were significantly improved.	[73]
C-Phycocyanin (*Spirulina platensis*)	The protein was modified using formaldehyde crosslinking	Increases photostability of modified protein only upon yellow light irradiation.	[74]
C-phycocyanin (*Arthrospira platensis* IFRPD 1182)	Freeze-dried maltodextrin and gum Arabic (fractions from 0 to 100%) were used as protein microencapsulation wall materials	Increased thermal stability of encapsulated protein, with high antioxidant properties.	[75]
Phycobiliprotein *(Palmaria palmata*)	Phycobiliprotein within liposome (soy lecithin) stabilized using polyethylene glycol adsorbed cellulose nanocrystals.	The encapsulated protein was stable below 60 ℃, above pH of 5.0, and against illumination.	[76]
C-Phycocyanin (Spirulina)	High-pressure processing treatment of the protein–whey protein and protein–carrageenan complexes at acidic pH	Protein’s complexations improved the color and, therefore, its storage stability under light exposure.	[77]
R-Phycoerythrin (*Porphyra haitanensis*)	Preparation of the various oligochitosan-modified protein complexes (OMPC) via the transglutaminase-catalyzed glycosylation reaction	Emulsifying stability, thermal stability, photostability, and pH stability of the OMPC were all significantly improved.	[54]
